# Death of Dr. John G. Westmoreland

**Published:** 1887-04

**Authors:** 


### DEATH OF DR. JOHN G. WESTMORELAND.

Dr. John G. Westmoreland, a brother of Dr. W. F. Westmoreland, died on the 3d of March, at the residence of his son, in this city, after a long and painful illness.

He was born in Jasper county, Georgia, in the year 1816, and was, at the time of his death, seventy-one years of age. When a small boy, his parents moved to Fayette county, where he obtained a rudimental education, subsequently completed at other schools of the State. He selected the profession of medicine as his life pursuit, and entered the Medical College of Georgia. While in attendance upon the college, he devoted himself assiduously to his studies. He graduated in 1841 with distinction. Soon thereafter he located in Pike county, where, in partnership with the late Dr. J. G. Caldwell, of this city, he entered the practice of medicine. It was in the year 1853 that he came to this city and made it his home.

In 1846 he married Miss Louisa Buchanan, who died in 1850, leaving two children, a son and daughter, to his care.

Being a man of popular address, he was soon a central figure in his new home. His attainments in medicine gave him an extensive and lucrative practice, while by his enterprising spirit and sound judgment he became an adviser and leader in every movement affecting the community in which he lived. Devoted to Atlanta, he omitted nothing which tended to make her a prosperous city. His hand was ever ready to advance her welfare.

In 1854 he conceived the project of a medical college in this city, and entered promptly and zealously upon the work of its establishment. It was not long before he had the gratification of seeing the Atlanta Medical College a prosperous institution, holding equal rank with the very best schools of the South. Much of his own capital was called into requisition in founding this college, and for many years he was one of its most stable supports and distinguished professors. In 1855 he founded THE ATLANTA MEDICAL AND SURGICAL JOURNAL, which he edited with an ability that gave it high standing with the medical profession throughout the United States.

In 1857 he was chosen to represent the county of Fulton in the Legislature of the State. During his term of service he made the reputation of a safe, able and progressive legislator. While always watchful of the local interests which he was elected to advance, he did not lose sight of the general welfare of the commonwealth. As a debater he was practical, logical and earnest, forgetting self for the subject under discussion and seeming ambitious only to enforce the policy that his mind and heart approved. Returning to his constituents, they received him as one who had discharged the duties of his position well, and he could have continued to represent this county had he not positively refused to accept the honor. It was his resolve to devote himself to his profession, the college and THE JOURNAL, and to this resolution he adhered until the war burst upon the land and his country called for true men to defend her cause. Accordingly he abandoned every personal interest and entered the Southern army as a private in the Fulton Dragoons. His eminence as a physician and surgeon was too well known to allow him to be left as a private soldier in the ranks. Consequently he was called to hospital service in Richmond, Va., where he added fame to that already won in his profession.

When peace was restored he returned to this city and devoted himself to the college, which was re-opened, and to THE JOURNAL, the publication of which he resumed. Both of these interests were soon in prosperous condition.

For several years he has suffered declining health.

He leaves only one child, Dr. Robt. W. Westmoreland, of this city. His funeral was attended by the physicians of Atlanta in a body and by many friends who knew him through life. His remains rest in Oakland Cemetery.

### ITEMS.

THE TEXAS HOMŒOPATHIC MEDICAL ASSOCIATION will be held in Fort Worth, May 3rd and 4th.

THE CHICAGO COLLEGE OF PHARMACY graduated forty-four applicants at their recent commencement.

DR. A. G. NORTH, a prominent physician of McDonough, Ga., gave THE JOURNAL a pleasant call a few days since.

DR. W. F. WESTMORELAND, JR., of this city, left for New York a few days since to be gone for several months.

SOMETHING NEW.—We have received a writing tablet from Wells & Richardson Co., the proprietors of lactated food, which is something new. It will be sent to any physician on application to them at Burlington, Vt., if this journal is mentioned.

THE AMERICAN SYSTEM OF GYNÆCOLOGY will soon be issued from the press of Lea Brothers & Co., Philadelphia. Among the prominent contributors we note Drs. Battey, Barker, Goodell, Reeves Jackson, Engelmann, Lusk, Munde, Reamy and a number of other equally prominent authorities.

JOEL CHANDLER HARRIS has contributed to the next number of *The Century* a story entitled "Little Compton," the scene of which is laid in Georgia before, during and at the close of the war. It is, in fact, a war story from the Southern point of view, and is characteristically illustrated by A. B. Frost.

PROFESSOR A. S. HILL, of Harvard, contributes to the April *Scribner* a short but comprehensive paper on "English in our Colleges," with some fresh and individual discussion of a subject which is always sure of attention from those who are interested in securing sound and useful higher education.

ANNALS OF SURGERY.—This excellent journal comes to us for March full of interesting and valuable matter for the surgeon. It is the only work of its kind published in this country, and deserves to be liberally patronized. It is published simultaneously in Great Britain and in the United States by J. H. Chambers & Co., St. Louis.

PERSONAL.—We regret to learn that Dr. Henry Wile, of this city, has been forced by ill health to abandon his practice for a time. He goes, on April 1st, to Denver, Colorado. We trust that he will soon be sufficiently restored to return and resume his work. No man stands higher in the profession in Atlanta than Dr. Wile. His many friends here and elsewhere will regret to learn of his bad health.

SOUTHERN MEDICAL COLLEGE COMMENCEMENT.—The Southern Medical College, of this city, held its annual commencement at DeGive's Opera House on the evening of March 3d. The annual address to the graduates was delivered by Rev. Henry Quigg, D. D., of Covington, and was well received by the large and intelligent audience. There were thirty graduates as follows: W. H. Aycock, J. A. S. Bailey, H. C. Bates, B. W. Bizzell, K. H. Boland, A. S. Bridwell, A. O. Brooks, M. M. Crowder, C. M. Curtis, J. A. Dobbins, B. W. Fite, W. L. Green, J. A. Ison, G. W. Julian, J. C. Kerr, M. H. Lee, T. D. McDaniel, W. A. Means, J. E. Miller, J. R. Mitchell, H. A. Mobley, A. J. Morris, C. T. Quinn, J. R. Reeve, D. D. Reid, J. T. Renouff, W. R. Rice, T. B. Robertson, D. M. Wheelis, C. M. Wyatt.

SUBSTITUTION BY DRUGGIST.—The *St. Louis Medical and Surgical Journal* for March, 1887, closes an editorial on this subject as follows: Substitution is not simply dishonest; it is felonious and displays the same reckless disregard for life that marks the burglar or highwayman who is prepared to take a life if it stands in the way of his plunder. The man who does it does not simply filch a few cents from the pocket of his customer (frequently poor and needy), nor does he merely jeopardize the reputation of a physician, but he puts in peril the life of the customer who trusts him.

The honest members of an honorable profession—and fortunately they are largely in the majority—the reputable pharmacists, owe it to themselves to expose these vultures and drive them from the trade. In doing so they should have the aid and countenance of every physician. In the meantime, let every physician not content himself with shunning the shops of those whom he detects in the nefarious habit of substitution, but boldly denounce them, and warn his patients against carrying prescriptions to them. Concerted action of this sort will soon purge the trade of the offending members.

REMOVAL OF THE LOWER JAW—RESULTS.—Dr. K. C. Divine, of this city, writes us under date March 21: "In the February number of your journal, page 760, is the report of giant cell sarcoma of the lower jaw, removed February 9th, 1886; and thinking that some of the many readers of your valuable journal would be pleased to know of her present condition, I am induced to send you the following: On the 15th instant she writes: 'I am well and enjoying good health; can articulate every letter of the alphabet perfectly except V; talk and sing almost as well as I ever could; do all my housework (seven in family, including a bright and perfectly-formed baby-girl eight months old on the 14th instant, and who is bountifully nourished from the source that nature supplies); can open and close my mouth without inconvenience. There is no dribbling, whether asleep or awake, and I am growing fat.' It may not be amiss to add that the birth of the child on the 14th of July, 1886, was a surprise to me, and I presume to the mother also, as no reply of hers to my many inquiries as to her health raised the slightest suspicion that she was pregnant, yet at the time of the operation she was over four months advanced in pregnancy. It is a surprise to those who were present at the operation and witnessed the severity of the shock and the vigorous measures resorted to for the purpose of reviving the patient, that the progress of utero-gestation should have proceeded with no disturbance or perceptible ill effects."

THE MEDICAL ASSOCIATION OF ALABAMA will meet in Tuscaloosa on April 12th and continue in session four days. This Association is the best organized medical body in the South, and is doing a great work in the State.

GEORGE A. SHEPARD, representing lactated food, a food for infants and invalids, manufactured in Burlington, Vermont, by Wells & Richardson Company, was distributing samples of this food among the physicians of Atlanta a few days since.

THE NEW PRINCETON REVIEW.—We are in receipt of the March number of this periodical. It has accustomed its readers to literary features of the most original and deeply interesting character, but it has certainly secured nothing more brilliant than Henri Taine's characterization of "Napoleon Bonaparte" in its March issue. A more incisive and splendid piece of writing has not been read for many a day than this study of a man of genius on the side of action by a man of genius on the side of reflection.

COMMENCEMENT OF THE ATLANTA MEDICAL COLLEGE.—The twenty-ninth annual commencement of this college was held in DeGive's Opera House, on the evening of March 4th, before a large and cultured audience. The report of the Proctor showed that there had been one hundred and twenty-one students in attendance during the season; fifty-six of these were second course; forty-five of this number received the degree. Following is a list of the graduates: J. L. Barge, Georgia; H. C. Baxter, Alabama; J. L. Byron, Georgia; M. W. Chambers, South Carolina; C. O. Champion, North Carolina; F. A. Christian, Georgia; B. F. Daniel, Georgia; J. H. Downey, Georgia; T. E. Drewry, Georgia; J. E. Farber, Florida; W. H. Godwin, Georgia; J. W. Hamilton, Georgia; E. A. Harris, Georgia; W. T. Herring, Georgia; B. B. Jameson, Georgia; J. C. Johnson, Georgia; S. W. Johnson, Georgia; F. M. Jones, Georgia; E. G. Lind, Jr., Georgia; F. L. Malone, Georgia; S. G. Noell, Georgia; A. D. Olds, Georgia; A. L. Payne, Georgia; S. B. Polland, Georgia; S.W. Pryor, South Carolina; J. H. Reeves, Georgia; J. B. Rhodes, Georgia; T. B. Rider, Tennessee; J. R. Ritch, Georgia; L. Roberts, Georgia; G. C. Roberts, Georgia; S. Rumble, Georgia; F. W. Runyan, Georgia; W. M. Ryals, Georgia; W. G. Sexton, South Carolina; W. G. Shipp, Georgia; L. P. Stephens, Georgia ; J. B. Stepp, South Carolina; H. B. Tate, South Carolina; J. H. Walton, Jr., Georgia; T. J. Wills, Georgia; R. J. Wilson, Georgia; W. J. Williams, Georgia; W. F. Yarbrough, Georgia; W. H. Young, Mississippi. The valedictory, by Dr. J. C. Johnson, of the class, was clothed in the most beautiful language and was well delivered. Rev. Henry Clay Morrison, of this city, delivered a most excellent address to the class. This address is published in full elsewhere in THE JOURNAL.

THE TREATMENT OF SYPHILIS.—At a recent discussion at the Medical Society of London on a paper read by Mr. Milner, divergent opinions with regard to the treatment of syphilis were expressed, and some valuable and interesting remarks were made by Dr. Lauder Brunton and Dr. Althaus. The effect of mercury in controlling to some extent the secondary manifestations of syphilis is assumed to be due to its properties as a germicide, and the same convenient explanation is given of the undoubtedly beneficial action of iodide of potassium in the later stages. It was pointed out, however, that the effects of iodide of potassium or sodium are due, not to the iodine, but to the salt which is not a germicide. Further, it was remarked by several speakers that the action of the iodides, while prompt and certain in dissipating the local symptoms which are supposed to belong to the later stages of syphilis, is not permanent. It has, indeed, been generally recognized that the iodides of potassium and sodium are only indicated in the lesions which occur in various tissues from the action of the virus in the past. Dr. Brunton appeared disposed to allow that mercury might owe its effect in syphilis to its action as a germicide, and observed that whatever its influence on the diseased germ, it was detrimental to the tissues. Further, there was a very general expression of opinion that iodide of potassium, like mercury, was not a drug to be trifled with, and the propriety of employing mercury, even in the later stages, found several supporters. Inunction as a method of treatment has become more systematic of late years, especially since pharmaceutical research has placed at our disposal more elegant and convenient preparations for the purpose. Dr. Althaus spoke very highly of the oleate of mercury as a cleanly and readily absorbed preparation, and the peculiar qualities of lanoline as a vehicle for the administration of drugs, and particularly mercury, by the skin, also elicited expressions of approval. For severe cases, the intra-muscular injection of mercury in some form or another, as practiced by Mr. J. Astley Bloxam at the Lock Hospital, is productive of good results, though patients are apt to resent the pain which accompanies its introduction. Each method of mercurial administration possesses certain advantages. A clear and well-defined classification of the indications for each method is still wanting, though Dr. Milner has filled the gap to some extent in the paper referred to.—*British Medical Journal, March 12, 1887.*

